# Nitric Oxide Induces Cardiac Protection by Preventing Extracellular Matrix Degradation through the Complex Caveolin-3/EMMPRIN in Cardiac Myocytes

**DOI:** 10.1371/journal.pone.0162912

**Published:** 2016-09-20

**Authors:** Irene Cuadrado, Borja Castejon, Ana M. Martin, Marta Saura, Paula Reventun-Torralba, Jose Luis Zamorano, Carlos Zaragoza

**Affiliations:** 1 Cardiology Department, University Francisco de Vitoria/Hospital Ramón y Cajal Research Unit (IRYCIS), Ctra. Colmenar Viejo, km. 9100, 28034, Madrid, Spain; 2 Department of Systems Biology (Physiology), University of Alcalá, School of Medicine (IRYCIS), Ctra. Madrid Barcelona, Km 3,300, 28875, Alcalá de Henares, Madrid, Spain; 3 Cardiology Department, University Hospital Ramón y Cajal (IRYCIS), Ctra Colmenar Viejo, km. 9100, 28034, Madrid, Spain; University of Louisville, UNITED STATES

## Abstract

Inhibition of Extracellular Matrix degradation by nitric oxide (NO) induces cardiac protection against coronary ischemia/reperfusion (IR). Glycosylation of Extracellular Matrix Metalloproteinase Inducer (EMMPRIN) stimulates enzymatic activation of matrix metalloproteinases (MMPs) in the heart, although the mechanisms leading to EMMPRIN glycosylation are poorly understood. We sought to determine if NO may induce cardiac protection by preventing glycosylation of EMMPRIN in a mouse model of IR. Here we found that Caveolin-3 binds to low glycosylated EMMPRIN (LG-EMMPRIN) in cardiac cells and in the hearts of healthy mice, whereas IR disrupted the complex in nitric oxide synthase 2 (NOS2) knockout (KO) mice. By contrast, the binding was partially restored when mice were fed with an NO donor (DEA-NO) in the drinking water, showing a significant reduction on infarct size (NOS2KO: 34.6±5 *vs* NOS2KO+DEA-NO: 20.7±9), in expression of matrix metalloproteinases, and cardiac performance was improved (left ventricular ejection fraction (LVEF). NOS2KO: 31±4 *vs* NOS2KO+DEA-NO: 46±6). The role of Caveolin-3/EMMPRIN in NO-mediated cardiac protection was further assayed in Caveolin-3 KO mice, showing no significant improvement on infarct size (Caveolin-3 KO: 34.8±3 *vs* Caveolin-3 KO+DEA-NO:33.7±5), or in the expression of MMPs, suggesting that stabilization of the complex Caveolin-3/LG-EMMPRIN may play a significant role in the cardioprotective effect of NO against IR.

## Introduction

MMPs are proteolytic degrading enzymes that cleave extracellular matrix (ECM) components. In the heart, MMP enzymatic activation induces cardiac myocyte necrosis, heart failure, and abnormal ventricular remodeling [1]. The Extracellular Matrix MetalloPRoteinase INducer EMMPRIN (CD147, Basigin) regulates the expression of several MMPs, including MMP-2 and MMP9, and it plays a pivotal role in the inflammatory response to ischemia in monocytes and cardiac cells [2, 3].

Caveolae are cholesterol and sphingolipid enriched small vesicles present in the cell membranes of several cell types, including cardiac myocytes [4]. Caveolae harbor many signaling pathways, in which caveolins play a dual role as structural, and regulatory elements through protein-protein interaction with resident caveolar proteins [5]. The expression patterns of Caveolin -1, and -2, are distinct from that of Caveolin-3, the later limited to smooth, skeletal and cardiac muscle [6].

The role of caveolins in the NO signaling pathway has been well documented. In the cardiovascular system Caveolin-1 and -3 interact with NOS3, resulting in eNOS inactivity [7]. However, in response to IR they both induce cardiac protection by different mechanisms, including those leading to NO production [8, 9].

EMMPRIN contains two extracellular Ig domains. The first domain is required for MMP activation in high-glycosylated EMMPRIN oligomers at the cell surface of many cell types [10]. We and others found that NO prevents the expression of EMMPRIN in cardiac myocytes [3, 11], but the precise role at the protein level is yet unknown. The second Ig domain of EMMPRIN is required for interaction with other proteins. Low glycosylated EMMPRIN (LG-EMMPRIN) binds to Caveolin-1, and prevents EMMPRIN glycosylation and self-aggregation, a requirement to induce MMP activation [10].

In the present work we investigated the contribution of NO in the interaction between Caveolin-3 and EMMPRIN during a IR, as a mechanism to prevent ECM degradation in cardiac myocytes.

## Materials and Methods

### Reagents

Evans blue, Triphenyl tetrazolium chloride (TTC), doxycycline and interleukin-18 (IL-18) were from Sigma (Spain). Horse radish peroxidase (HRP)-conjugated anti-mouse secondary antibody and liquid 3,3'-diaminobenzidine (DAB) substrate were from Dako (Carpinteria, CA). Anti MMP-9, anti Caveolin-3, anti- and FITC-conjugated secondary antibodies were from Santa Cruz Biotechnology (Santa Cruz, CA). HRP-conjugated anti-rabbit secondary antibody, was from Sigma-Aldrich. Amersham ECL detection kit was from GE (GE Healthcare Life Sciences, Spain). Centrifugation concentrators were from Sartoriuos (Fischer Scientific, Spain).

### Animals

NOS2 null mice, Caveolin-3 null mice, and the corresponding wild-type controls were housed in our animal facilities in isolated rooms. Animal studies have been performed in accordance with the ethical standards laid down in the 1964 Declaration of Helsinki and its later amendments. All animal procedures were approved by the National Research Ethics Committee and conformed to EU Directive 86/609/EEC and recommendation 2007/526/EC regarding the protection of animals used for experimental and other scientific purposes (enacted under Spanish law 1201/2005).

### Cells

The HL1B cell line was kindly donated by Dr. Antonio Bernad. Cells were cultured in T150 flasks with Claycomb media, supplemented with 10% FCS, 0.1mM norepinephrine, 1.5 mM L-glutaminie and 50 units/ml penicillin-streptomycin. After confluence, cells were split into 10 cm dishes for experimentation.

### Animal model of Ischemia/Reperfusion

Ischemia was induced by coronary artery ligation. Twelve-week- old mice were intraperitoneally anesthetized with ketamine/xylazine (100 and 10 mg/kg, respectively), intubated with a 1-mm steel tube, and ventilated (2ml, 80 strokes/min). The thorax was opened between the second and the third ribs and widened with the aid of a mouse retractor. The pericardium was opened and the left anterior descending (LAD) coronary artery was occluded for 30 min close to its bifurcation with a 6–0 silk suture. Reperfusion was performed by releasing suture, 30 minutes after LAD occlusion. The chest was closed, negative pressure was reestablished, and the skin was sutured. Control mice (sham) were included in the assays, in which the same procedure was performed except for LAD occlusion.

### Determination of infarct size

Myocardial infarct size was determined by double staining with Evans Blue/TTC dyes. Animals were anesthetized and a 0.2% Evans Blue solution was injected into the aorta allowing uniformly distribution of the dye. Animals were sacrificed, the hearts frozen, sliced in 1mm ring sections perpendicular to the LV long axis, and incubated with 0.5% TTC solution for 5 minutes. Healthy tissue (blue), area at risk (red), and infarct tissue (pale white) areas were subjected to morphometric analysis by using the Motic Images Plus software.

### Echocardiography

Mouse hearts were visualized by echocardiography, using a ultrasound system (Vivid Q, GE). During all experiments, mice were anesthetized with 1.5% isoflurane gas, resulting in a heart rate of approximately 400 beats/min. The chest of the mice were carefully shaved, and warm ultrasound transmission gel was applied to ensure optimal image quality. Parasternal short-axis-view images of the heart were recorded n a B-mode to allow M-mode recordings by positioning the cursor in the parasternal short-axis view perpendicular to the inter-ventricular septum and posterior wall of the left ventricle. From these recordings, the following parameters were determined using the on-site software cardiac package: left-ventricle end-diastolic diameter, left-ventricle end-diastolic volume, ejection fraction, and shortening fraction.

### Histology and immunohistochemistry

Histological and immunohistochemical procedures were performed as previously described [12].

### Immunoblot analysis

Isolation of protein lysates and immunoblots were performed as described [12].

### Discontinuous sucrose gradient

Total hearts were isolated and washed twice in ice-cold PBS. Heart tissues were homogenized in a buffer containing 1 ml of 150 mM sodium carbonate/1 mM EDTA pH 11.0, following 10 times of 5 second bursts in a tissue grinder, combined with 15 seconds sonication, and 1 min resting on ice. Protein concentration was adjusted to 0.5 mg/ml and mixed with 1 ml of 80% sucrose in (MES)-buffered saline (MBS). The solution was separated by ultracentrifugation in a discontinuous sucrose gradient (40%-30%–5%) in a SW40 rotor (Beckman) at 200,000 × *g* for 18 h. After centrifugation, 1ml fractions were collected, starting at the upper part of the centrifuge tube (lower buoyant density). Samples were separated by SDS-PAGE, and subjected to immunoblot with anti-Caveolin-3 and anti-NOS2 antibodies.

### Statistical analysis

Unless otherwise specified, data are expressed as means SD. Cell culture experiments were performed in triplicate, and conditions were assayed in duplicate on each replicate. Animal experiments were performed in triplicate, and the numbers of animals and replicates are specified in the text. Whenever comparisons were made with a common control, significance of differences was tested by analysis of variance followed by Dunnett's modification of the t test. Differences were considered significant at pb0.05. Error bars represent ± SD.

## Results

### Ischemia/Reperfusion induces the expression and glycosylation of EMMPRIN in NOS2 KO mice

**Fig 1:**
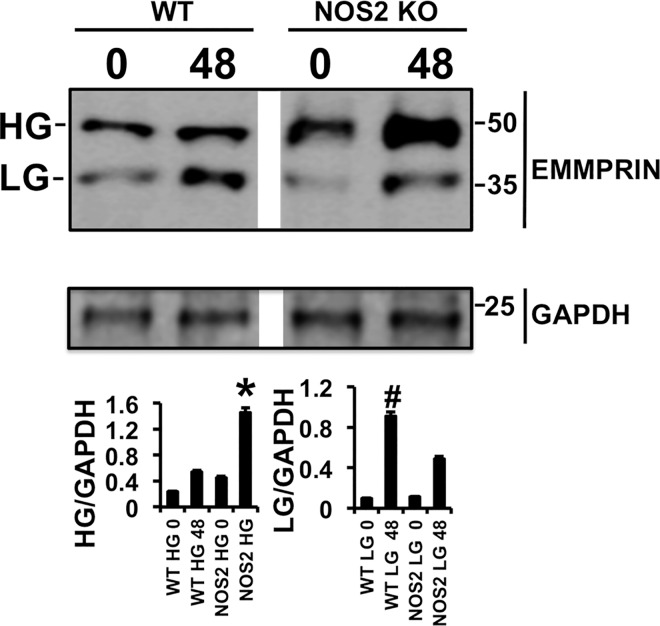
Ischemia/Reperfusion induces the expression and glycosylation of EMMPRIN in NOS2 KO mice. Immunoblot detection of EMMPRIN in WT and NOS2 KO mice 48 hours after IR. GAPDH was used as loading control. HG: high glycosylated EMMPRIN. LG: Low glycosylated EMMPRIN. (n = 9 mice, mean ± SD *p<0.05 WT HG 48 vs NOS2 HG 48. #p<0.05 WT LG 48 vs NOS2 LG 48).

IR induces the expression of EMMPRIN in the hearts of WT and NOS2 KO mice, as detected 48 hours after reperfusion (Fig 1). Two fragments of 35 kDa and 50 kDa, corresponding to low glycosylated (LG) and high glycosylated (HG) EMMPRIN were detected by immunoblot. However, the ratio between HG-EMMPRIN/LG-EMMPRIN of 0.4 in WT mice was shifted to 2.8 in NOS2 KO mice, suggesting that in response to IR, glycosylation of EMMPRIN was stimulated in the absence of NO (Fig 1).

### Caveolin-3 binds to EMMPRIN in cardiac myocytes

**Fig 2:**
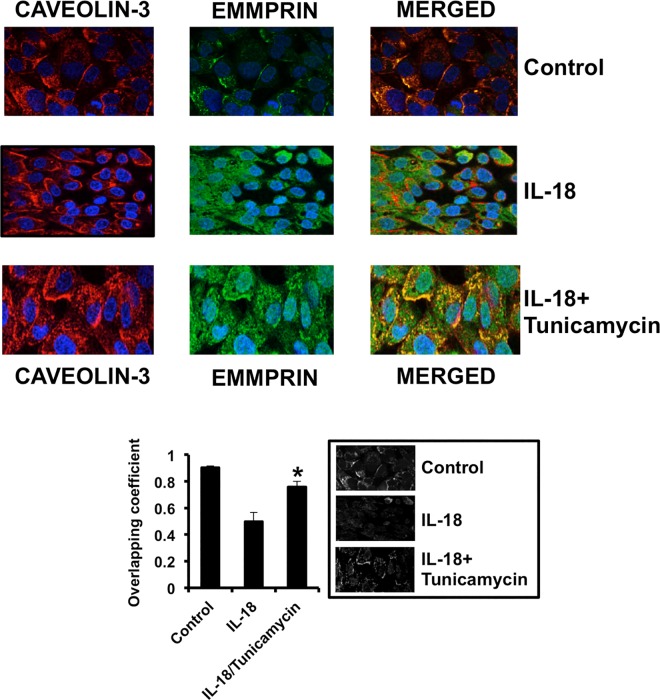
Glycosylated EMMPRIN binds to Caveolin-3 in cardiac cells. Confocal microscopy detection of Caveolin-3 (Cy-3, red) and EMMPRIN (FITC, green) in HL1B resting (upper panels), incubated 8 hours with 100 μM IL-18 (middle panels), or with 100 μM IL-18 plus 10 μM tunicamycin (lower panels). Colocalization of both signals is detected in merged panels (yellow) (n = 9 plus triplicates). Bottom left: Co-localization analysis as detected by calculation of overlapping correlation coefficient ((n = 9 plus triplicates. *p<0.05 IL-18 vs IL-18+tunicamycin). Bottom right: Micrographs corresponding to one representative assay in which overlapping green and red signals are highlighted in white.

We found that EMMPRIN co-localizes with Caveolin-3 in resting HL1B cardiac myocytes, as detected by confocal microscopy (Fig 2, Control). Incubation of HL1B cells with 100 μM IL-18 induced the expression of EMMPRIN, and a significant inhibition in the binding to Cavelolin-3 (Fig 2 IL-18). To test whether EMMPRIN glycosylation may play a role, co-incubation with 100 μM IL-18 plus 10 μM tunicamycin, a pharmacological inhibitor of N-glycosylation of proteins, restored the complex formation back to control cells (Fig 2, IL-18/Tunycamycin). Taken together, these data suggest that Caveolin-3 binds to LG-EMMPRIN.

### IR disrupts the complex Caveolin-3/EMMPRIN in the heart

**Fig 3:**
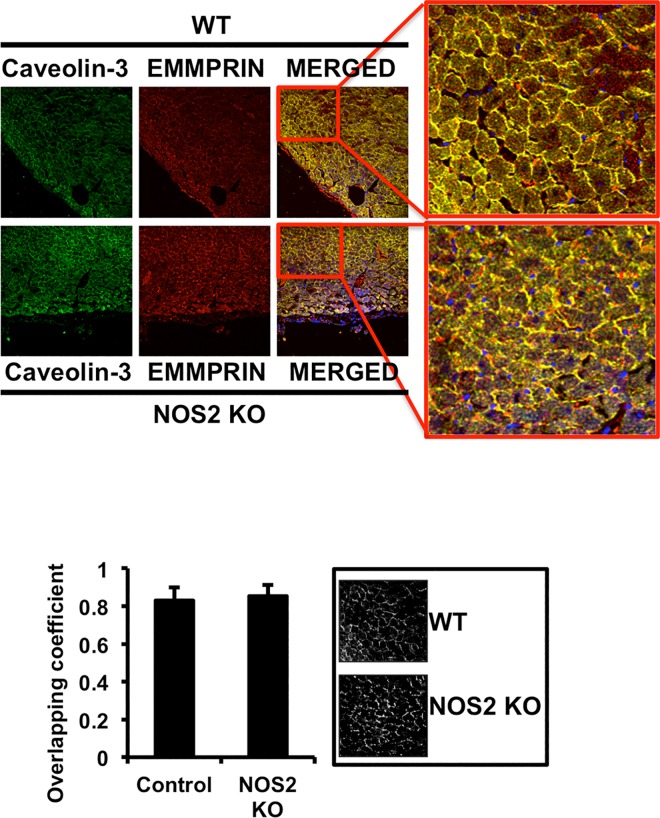
EMMPRIN co-localizes with Caveolin-3 at the cell surface of healthy mouse hearts. Confocal microscopy detection of Caveolin-3 (FITC, green), and EMMPRIN (Cy3, red) in heart sections from healthy WT and NOS KO mice with specific antibodies. Co-localization (yellow) is shown in the merged panels (n = 9 mice/strain/triplicated). Bottom left: Co-localization measurement as detected by calculation of overlapping correlation coefficient ((n = 9 plus triplicates). Bottom right: Micrographs corresponding to one representative assay in which overlapping green and red signals are highlighted in white.

**Fig 4:**
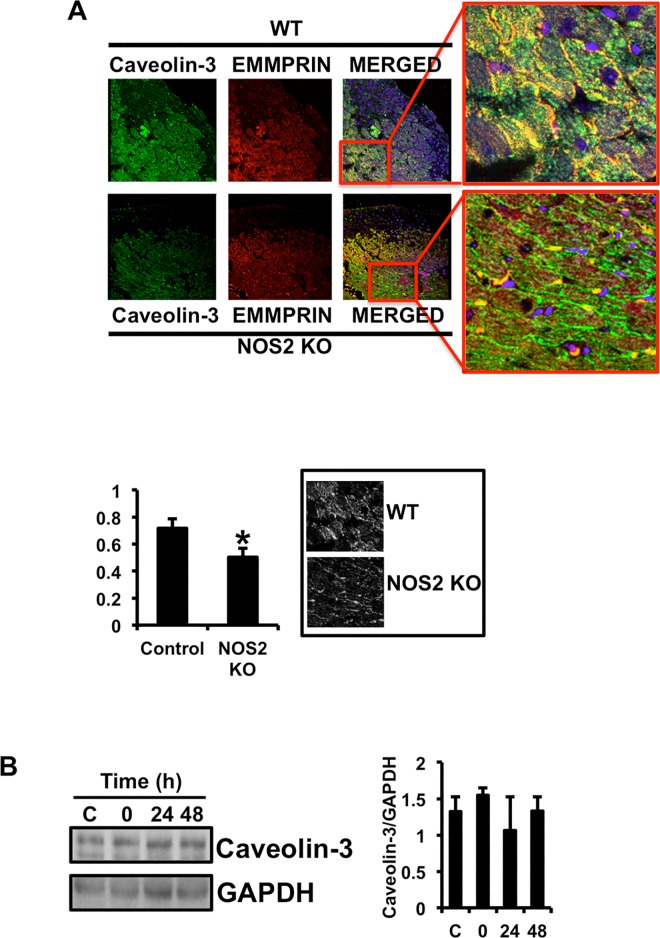
Ischemia/Reperfusion disrupts the complex Caveolin-3/EMMPRIN in mouse hearts. (A) Confocal microscopy detection of Caveolin-3 (FITC, green), and EMMPRIN (Cy3, red) in heart sections from WT and NOS KO mice 48 hours after IR with specific antibodies. Co-localization (yellow) is shown in the merged panels (n = 9 mice/strain/triplicate). Bottom left: Co-localization measurement as detected by calculation of overlapping correlation coefficient ((n = 9 plus triplicates. *p<0.05 Control vs NOS2 KO). Bottom right: Micrographs corresponding to one representative assay in which overlapping green and red signals are highlighted in white. (B) Left. Immunoblot detection of Caveolin-3 from heart lysates after IR at the times indicated. The expression of GAPDH was used as loading control. Right. Densitometric analysis of the bands corresponding to the expression of Caveolin-3 in reference to the levels of GAPDH.

Caveolin-3 co-localizes with EMMPRIN in WT and NOS KO mice, as detected by confocal microscopy of heart sections immunostained with specific antibodies (Fig 3). By contrast, IR significantly reduced co-localization in NOS2 KO respect to WT mice (Fig 4A). Since IR did not reduced the levels of Caveolin-3 (Fig 4B), our data suggest that lack of NO in vivo promotes the dissociation of the complex Caveolin-3/EMMPRIN in response to IR.

### The disruption of the complex Cavolin-3/LG-EMMPRIN was increased in the absence of NOS2

**Fig 5:**
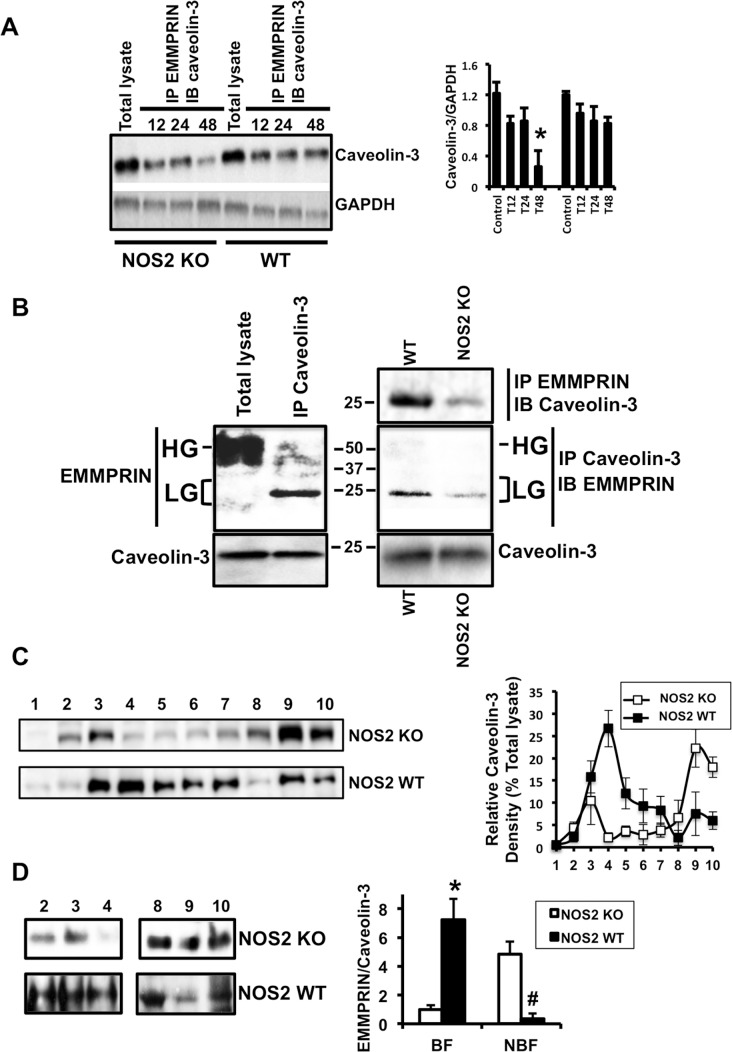
The complex Caveolin-3/EMMPRIN is disrupted in NOS2 KO mice 48 hours after Ischemia/Reperfusion. (A) Immunoblot (IB) detection of Caveolin-3 from immunoprecipitated extracts (IP) with anti-EMMPRIN antibody, isolated from heart lysates of mice under IR at the times indicated. Immunoblot detection of GAPDH was used as loading control (n = 9 mice, mean ± SD *p<0.05 WT vs NOS2 48h). (B) Upper-Left panel. Immunoblot detection of EMMPRIN from total lysates or immunoprecipitated extracts with anti-Caveolin-3, and isolated from mouse hearts 48 hours after IR. Bottom-left panel. Immunoblot of Caveolin-3 from the same extracts as above. Upper-right panel. Immunoblot detection of Caveolin-3 from immunoprecipitated extracts with anti-EMMPRIN and isolated from WT and NOS2 KO hearts isolated 48 hours after IR. Middle-Right panel. Immunoblot detection of EMMPRIN from immunoprecipitated extracts with anti-Caveolin-3, from the same mice as before. Lower-Right panel. Immunoblot detection of Caveolin-3 from total cell lysates and immunoprecipitated with anti-Caveolin-3 from the same mice as before (n = 9 mice by triplicate). (C) Immunoblot detection of Caveolin-3 in WT and NOS2 KO heart proteins separated by discontinuous sucrose gradient fractionation. Right panel. Densitometric analysis of Caveolin-3 distribution in WT and NOS2 KO fractions. (n = 3 mice by triplicate, mean ± SD). (D) Immunoblot detection of LG-EMMPRIN in buoyant (2, 3, 4) and non buoyant (8, 9, and 10) fractions from WT and NOS2 KO heart proteins separated by discontinuous sucrose gradient fractionation (n = 3 mice by triplicate, mean ± SD *p<0.05 WT vs NOS2 BF; ^#^p<0.05 WT vs NOS2 NBF).

High glycosylated EMMPRIN oligomerize in the cell surface of many cell types and induces enzymatic activation of several MMPs. To test whether IR could regulate glycosylation of EMMPRIN, we first immunoprecipitated heart protein lysates isolated 12h, 24h, and 48 hours after IR with anti-EMMPRIN antibodies (Fig 5A). EMMPRIN immunoprecipitated extracts contained similar amounts of Caveolin-3 excepting 48 hours after IR, in which a significant reduction of Caveolin-3 was detected in NOS2 KO mice, when compared to the levels found in WT animals (Fig 5A). On the other hand, in Caveolin-3 immunoprecipitated extracts, mainly LG-EMMPRIN was present in the hearts of WT mice (Fig 5B, left panels), containing 4 times more LG-EMMPRIN, compared to the levels found in NOS2 KOs (Fig 5B, right panel), implying that NO may prevent glycosylation of EMMPRIN, by preserving the complex Caveolin-3/LG-EMMPRIN in response to IR.

Caveolin-3 exerts its biological function at the time it presents in buoyant membranes. We show that in NOS2 KO heart protein lysates subjected to discontinuous sucrose gradient fractionation, Caveolin-3 distribution shifted from fractions of lower (buoyant fractions (BF) 2, 3, 4) to higher (non buoyant fractions (NBF) 8, 9, and 10) density (Fig 5C), whereas LG-EMMPRIN was mostly concentrated in non-buoyant fractions in NOS2 KO mice. Taken together, our results suggest that in NOS2 expressing mice, Caveolin-3 may inhibit EMMPRIN glycosylation by forming a complex in buoyant membranes (Fig 5D).

### NOS2-induced cardiac protection is mediated by preserving the complex Caveolin-3/LG-EMMPRIN

**Fig 6:**
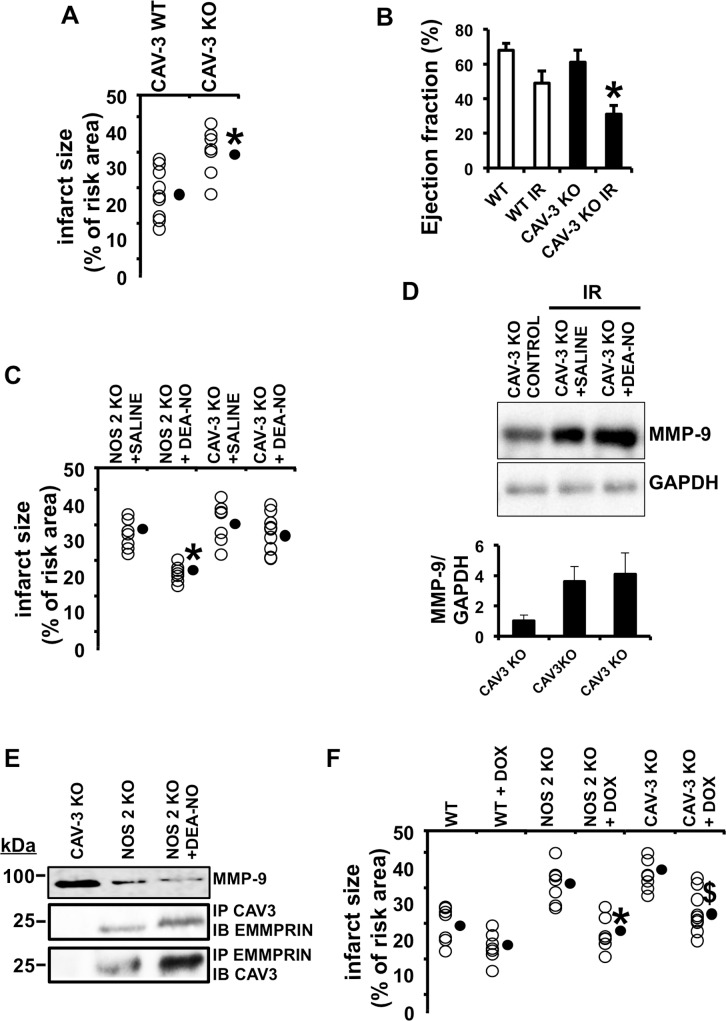
NOS2 induces cardiac protection through the binding of Caveolin-3 with LG-EMMPRIN. (A) Infarct size of WT and Caveolin-3 KO hearts 48 hours after IR, as detected by double Evans Blue/TTC staining (n = 9 mice/group; mean ± SD; *p <0.05) (B) LVEF in the same mice as before (mean ± SD; *p <0.05, WT IR vs Caveolin-3 KO IR). (C) Infarct size 48 hours after IR of NOS2 KO and Caveolin-3 KO mice and infused with saline or DEA-NO (n = 9 mice/group; mean ± SD; *p <0.05 NOS2 KO saline vs NOS2 KO DEA-NO). (D) Upper panel. Immunoblot detection of MMP-9 from total protein lysates isolated from Caveolin-3 KO mice and Caveolin-3 KO mice treated with saline or DEA-NO, 48 hours after IR. The expression of GAPDH was used as loading control. (n = 9 mice/group; mean ± SD). (E) Upper-panel. Immunoblot detection of MMP-9 from total protein lysates isolated from Caveolin-3 KO, NOS2 KO and NOS2 KO mouse hearts treated with DEA-NO. Middle-panel. Immunoblot detection of EMMPRIN from immunoprecipitated extracts with anti-Caveolin-3, from the same mice as before. Lower-panel. Immunoblot of Caveolin-3 from immunoprecipitated extracts with anti-EMMPRIN, from the same mice as before (n = 9 mice by triplicated). (F) Infarct size of WT, NOS2, and Caveolin-3 KO hearts 48 hours after IR, and previously fed with 50 mg/kg doxycycline for 1 week (n = 9 mice/group; mean ± SD; *p <0.05 NOS2 KO vs NOS2 KO + DOX. ^$^p <0.05 CAV3 KO vs CAV3 KO + DOX).

To assess whether NOS2 may induce cardiac protection by promoting the binding between Caveolin-3 and LG-EMMPRIN, we found that in Caveolin-3 KO mice subjected to IR, infarct sizes (Fig 6A) and left ventricle ejection fractions (LVEF, Fig 6B) were significantly higher than in Caveolin-3 expressing mice. Interestingly, exogenous administration of the NO donor DEA-NO in the drinking water notably reduced the infarct size in NOS2 KO mice, while DEA-NO had no effect in Caveolin-3 KO mice under IR (Fig 6C), implying a role of Caveolin-3 in the cardioprotective effect of NO in response to IR.

To further investigate the contribution of Caveolin-3 on EMMPRIN glycosylation, and the downstream EMMPRIN-mediated MMP activation, we found that IR induced the levels of MMP-9 in Caveolin-3 KO mice, and exogenous administration of DEA-NO had no significant effect (Fig 6D). By contrast, in NOS2 KO mice, the NO donor reduced the levels of MMP-9 in the hearts subjected to IR (Fig 6E upper panel), and the levels of the complex Caveolin-3/LG-EMMPRIN resulted increased, when compared to those found in NOS2 KO mice (Fig 6E, middle and lower panels). To further investigate the relevance of MMP enzymatic activity as a target of NO in cardiac protection, we administered 50mg/Kg doxycycline, a well known tetracycline derivative MMP inhibitor in the drinking water of WT, NOS2 KO, and Caveolin-3 KO mice, 1 week before IR (Fig 6F), detecting that in NOS2 KO and in Caveolin-3 KO mice, the lesions were significantly reduced. Taken together, our results suggest that NO induces cardiac protection at least in part, by decreasing ECM degradation through the preservation of the complex Caveolin-3/LG-EMMPRIN in response to IR.

## Discussion

In the current work we show new evidence about the molecular mechanism of NO in cardiac protection in response to IR. Our data support the hypothesis that NO protects the heart against IR by preventing ECM degradation. In resting conditions, we show for the first time that low glycosylated EMMPRIN is bound to Caveolin-3 in WT and NOS2 KO mice, while the complex was significantly reduced 48 hours after IR in NOS2 KO mice. Exogenous administration of DEA-NO to NOS2 KO mice rescued in part the WT phenotype, improving [heart function, and reducing the infarct size and the expression of MMP-9 in response to IR, whereas the same treatment failed to show any effect in Caveolin-3 KO mice. We suggest that the complex Caveolin-3/LG-EMMPRIN in NOS2 expressing mice is part of the mechanisms elicited by NO in cardiac protection.

The expression of EMMPRIN in response to acute myocardial infarction (AMI) has been described in monocytes/macrophages, in human cardiac myocytes and in animal models of IR [3, 13, 14]. We and others have found that inhibition of EMMPRIN improves cardiac function and reduces IR injury [15], but no data so far describes the mechanism that may regulate NO-mediated inhibition of EMMPRIN activity.

Caveolin proteins play a major role in the pathophysiology of several cardiovascular diseases [16, 17]. Caveolin-1 has been shown to induce cardiac protection against IR [16, 18–20], and recent evidences point towards Caveolin-1 as a target to prevent EMMPRIN glycosylation, by forming a complex with LG-EMMPRIN. Tang et al, have shown that silencing Caveolin-1 expression by siRNA, promoted the shift of HG-CD147/LG-CD147 ratio, from 2.1 in mock-treated cells, to 6 in Caveolin-1 silenced cells [10]. In the current work, we found a complex between Caveolin-3 and LG-EMMPRIN, suggesting that Caveolin-3 may also inhibit EMMPRIN glycosylation in a similar way. To our knowledge, Caveolin-3 protects the heart during anesthetic-induced cardiac preconditioning [21, 22], and it was recently found the role of Caveolin-3 in restoring Akt signaling in diabetic rats under IR [23]. Here, we show for the first time a role of Caveolin-3 in the NO-mediated cardiac protection, by forming a complex with low glycosylated forms of EMMPRIN.

The activation of MMPs is regulated by HG-EMMPRIN self-aggregation, [24, 25]. Caveolin-1 binds to LG-EMMPRIN and inhibits self aggregation by decreasing glycosylation of EMMPRIN in several cell types, including macrophage cell cultures [24]. We show here evidence about the role of Caveolin-3 as a target of NO in forming a complex with LG-EMMPRIN in mouse hearts, and the effect on cardiac function in the presence and in the absence of Caveolin-3, suggesting that Caveolin-3 prevents HG-EMMPRIN self aggregation, and thereby induces cardiac protection against IR injury.

We previously described that NO induces transcriptional inhibition of EMMPRIN [3]. To our knowledge no data regarding protein-protein binding domains between Caveolin -1, or -3 and EMMPRIN has been reported, although the Caveolin scaffolding domain (CSD) may be implicated, since many proteins bind to Caveolin -1, -3 through the CSD, including MMP-13, MMP-9, and endothelial nitric oxide synthase (eNOS, NOS3) [5, 7, 26], and the use of CSD peptides of Caveolin-1 and -3 regulate cardiac protection, and inhibit cardiac apoptosis [27].

Further studies focused to define the molecular mechanisms by which NO stabilizes the complex Caveolin-3/LG-EMMPRIN in response to IR will be required to precisely understand the molecular signaling pathways triggered by NO in cardiac protection. To this regard, the S-Nitrosylation of EMMPRIN and/or Caveolin-3, should be explored, since S-Nitrosylation have been suggested to induce cardioprotection against IR by still unknown mechanisms [28], and others have demonstrated that EMMPRIN gets S-Nitrosylated in the Golgi apparatus of endothelial cells [29], suggesting that in cardiac myocytes, NO which is highly produced in response to IR [3], may also induce S-Nitrosylation of EMMPRIN, as a mechanism of cardiac protection.
